# Orbital bistatic radar observations of asteroid Vesta by the Dawn mission

**DOI:** 10.1038/s41467-017-00434-6

**Published:** 2017-09-12

**Authors:** Elizabeth M. Palmer, Essam Heggy, Wlodek Kofman

**Affiliations:** 1 Department of Geosciences, Western Michigan University, 1903 W. Michigan Avenue, Kalamazoo, MI 49008-5241 USA; 2 Ming Hsieh Department of Electrical Engineering, University of Southern California, 3737 Watt Way, Los Angeles, CA 90089 USA; 3 Jet Propulsion Laboratory, California Institute of Technology, 4800 Oak Grove Dr., Pasadena, CA 91109 USA; 4 UGA/CNRS, Institut de Planétologie et d’Astrophysique de Grenoble (IPAG) UMR 5274, Grenoble, F-38041 France; 5 Space Research Centre of Polish Academy of Sciences, Bartycka 18A, 00-716 Warsaw, Poland

## Abstract

We present orbital bistatic radar observations of a small-body, acquired during occultation by the Dawn spacecraft at asteroid Vesta. The radar forward-scattering properties of different reflection sites are used to assess the textural properties of Vesta’s surface at centimeter-to-decimeter scales and are compared to subsurface hydrogen concentrations observed by Dawnʼs Gamma Ray and Neutron Detector to assess potential volatile occurrence in the surface and shallow subsurface. We observe significant differences in surface radar reflectivity, implying substantial spatial variations in centimeter-to-decimeter-scale surface roughness. Our results suggest that unlike the Moon, Vesta’s surface roughness variations cannot be explained by cratering processes only. In particular, the occurrence of heightened hydrogen concentrations within large smoother terrains (over hundreds of square kilometers) suggests that potential ground-ice presence may have contributed to the formation of Vesta’s current surface texture. Our observations are consistent with geomorphological evidence of transient water flow from Dawn Framing Camera images.

## Abstract (web summary)

The Dawn spacecraft has provided orbital bistatic radar observations of a small body in the solar system. Here, the authors present results from Vesta suggesting that smooth terrains with heightened hydrogen concentrations indicate that ground-ice presence potentially helped shape Vesta’s current surface texture.

## Introduction

Using the communications antenna aboard the NASA Dawn spacecraft, we conducted the first orbital bistatic radar (BSR) observations of a small body at asteroid Vesta at grazing incidence angles during entry and exit from occultations. In this configuration, Dawn’s high-gain telecommunications antenna (HGA) transmitted X-band radio waves during its orbit of Vesta, while the Deep Space Network (DSN) 70-meter antennas received the signal on Earth. Dawn’s orbital trajectory was designed to ensure that the spacecraft’s HGA communications antenna would almost constantly be in the line of sight with ground stations on Earth^1^, but on occasion, the spacecraft inevitably passed into occultation behind Vesta—lasting as briefly as 5 min or as long as 33 min. By continuously transmitting basic telemetry data from Dawn’s antennas during these events, the opportunity arose to observe surface reflections of HGA-transmitted radar waves from Vesta’s surface.

Over the past decades, orbital BSR experiments have been used to assess the textural (and in some cases, dielectric) properties of the surfaces of terrestrial bodies such as Mercury^2^, Venus^3^, the Moon^4–6^, Mars^7, 8^, Saturn’s moon Titan^9^, and now comet 67P/CG^10^. In contrast to most orbital BSR experiments, Dawn’s HGA beam intersected Vesta’s surface at grazing angles of incidence near 89° and in a microgravity environment with substantial variability in its gravity field^11^, leading to a low and variable orbital velocity and hence a more challenging detection of the Doppler-shifted surface echo (hereafter simply referred to as the “surface echo”). While the Mars Global Surveyor (MGS), for instance, orbited at ~3,400 m s^−1^ with respect to Mars’ rotating surface during its BSR experiment, Dawn orbited Vesta at a relative velocity of only ~200 m s^−1^. A high orbital velocity like that of the MGS results in a large Doppler shift between surface-reflected echoes and the direct signal, which greatly simplifies the need to distinguish the two peaks during spectral analysis. In the case of Dawn’s BSR observations of Vesta, however, much shorter averaging time and higher frequency resolution is needed to distinguish surface echoes^12^.

Through radar power spectral signal analysis of each surface echo, we assess relative surface roughness at centimeter-to-decimeter scales on Vesta and address its application to understanding the textural evolution of the surface. This is accomplished by measuring the radar cross section *σ* of each area that is illuminated by the radar lobe of Dawn’s HGA (hereafter referred to as the “site” or the “echo site”), which quantifies the cross-sectional surface area that—if equally scattering in all directions—would reflect the echo power measured at the receiver. Hence, larger values of *σ* are associated with stronger echoes. In turn, echo strength depends on the roughness of the surface at wavelength scales, the angle of incidence, and the intrinsic reflective and absorptive (dielectric) properties of Vesta’s surface material at radar wavelengths. Assuming each surface echo is measured at the same angle of incidence and is reflected from equal surface area, we normalize *σ* to the site of strongest reflection and use estimated dielectric properties of the surface material to assess relative centimeter-to-decimeter-scale surface roughness on Vesta with respect to a given reference site.

Through the comparison of relative surface roughness with estimated surface ages of geologic units based on two crater counting methods^13^, we assess the physical processes that shaped Vesta’s surface roughness at the same scales as done previously for the Moon (e.g., ref. ^14^). In turn, surface roughness provides insight into the shock history of the body^15^ and identification of fracturing mechanisms, such as those resulting from thermal erosion caused by diurnal expansion and contraction of volatiles within the surface host rocks^16^. The comparison of relative roughness with observations of hydrogen concentration [H] from Dawn’s Gamma Ray and Neutron Detector (GRaND^17^); with hydrated material distribution from Dawn’s Visible and Infrared Mapping Spectrometer (VIR^18^); and with surface thermal inertia and associated multi-meter-scale topography modeled from VIR data^19^ enable further investigation into the relationship between volatile presence and centimeter-to-decimeter-scale surface roughness. Characterizing the roughness properties of Vesta’s surface and of other small bodies is also key to assessing landing, anchoring, sampling, and surface trafficability in future missions^20^.

Given the low risk, low operational constraints, and opportunistic nature of the orbital BSR experiment by Dawn, other planetary missions can conduct similar observations even in the case of unlikely major science payload failure. Orbital BSR can also be used to constrain ambiguities associated with surface roughness and, potentially, surface dielectric properties of other small bodies as is proposed for the Jupiter Icy Moons Explorer (JUICE) mission at Ganymede^21^. In our study of Dawn’s BSR observations of asteroid Vesta, we successfully detect radar echoes from Vesta’s surface and find significant variations of radar reflectivity across the surface—where stronger surface echoes suggest smoother surfaces. Unlike the Moon, however, Vesta’s surface roughness variations cannot be explained by cratering only—particularly where smoother areas overlap areas of heightened [H], suggesting that volatile-involved processes have also contributed to shaping the surface of Vesta.

## Results

### Observation geometry and measurement constraints

During the BSR experiment at Vesta, the HGA aboard Dawn is used to transmit telemetry data at X-band radar frequency (8.435 GHz, 3.55 cm wavelength) while the three 70-m DSN antennas at Goldstone (USA), Canberra (Australia), and Madrid (Spain)—which have similar receiving characteristics (Supplementary Table 1)—are used to receive (Supplementary Fig. 1)^22^. Throughout the Dawn mission, the HGA continuously transmits right-hand circularly polarized (RCP) radio waves with a beamwidth of ~1.6°. The transmission frequency of the HGA is typically driven by the highly stable DSN uplink signal, as this allows for accurate Doppler and range tracking measurements^1, 11, 22^. When the uplink signal is not available—as anticipated in the minutes preceding and following an occultation of Dawn behind Vesta—the transmission frequency is instead driven by an internal auxiliary oscillator on board the spacecraft^1, 22^. While the onboard oscillator generates too much Doppler noise to be used for gravity science to measure the absolute Doppler shift of the direct signal^23^, its frequency is sufficiently stable over the integration time of BSR measurements—a few seconds rather than one minute—to measure the relative Doppler shift between the surface echo and direct signal, which are equally affected by slow Doppler changes. Due to the opportunistic nature of the experiment, the HGA also remains in a fixed orientation pointed toward Earth throughout each BSR observation^1^. As a consequence, Dawn’s transmitted radio waves scatter from Vesta’s surface just before and after each occultation of the Dawn spacecraft behind Vesta, resulting in surface echoes at high grazing incidence angles of ~89°.

The spacecraft’s trajectory is also designed to ensure that Dawn’s solar panels are constantly illuminated by sunlight. This geometry allows the primary observation instruments to have maximized visibility of Vesta’s sunlit surface throughout each orbit^1^. As a consequence, while Dawn is in a polar orbit around Vesta^24^, the sites intercepted by the spacecraft’s HGA beam yield surface echoes at mid-latitudes between 30°S and 45°N (Supplementary Fig. 2).

In contrast to previous planetary BSR observations performed for large bodies, with incidence angles between ~0° and ~80°, the surface reflections from Dawn’s BSR experiment are almost entirely in the regime of forward scattering by which the polarization of a circularly transmitted wave is conserved in major part even after reflection from the target’s surface (e.g., ref. ^25^). As a consequence, we cannot employ the typical method of measuring the circular polarization ratio to evaluate surface roughness or the dielectric constant from surface echoes, e.g., ref. ^26^, and instead develop a method to derive relative surface roughness by measuring the relative strength of reflected power from each echo site.

While DSN station operators record receiver system temperatures as part of standard calibration procedures^26^, this information was not included with the raw BSR data set, as these were acquired during the downlink of engineering-only telemetry. Since radar data acquired by the DSN are not calibrated in absolute voltage, we instead calibrate the received power to theoretical received power derived from known orbital geometry, and transmitter and receiver specifications. While each of the 70-m DSN antennas are held to the same measurement requirements (listed in Supplementary Table 1), we observe a decrease of ~10% in the direct signal’s ratio of measured to theoretical power over the course of a 33-min occultation, and differences in the ratio by as much as 24% from orbit to orbit. Fluctuations in the received power are attributed to variations in the pointing accuracy of the HGA aboard Dawn, since one of the four reaction wheels aboard the spacecraft—used to counteract pointing errors—failed prior to rendezvous with Vesta. We minimize the effect of variable pointing accuracy by measuring the direct signal within a few seconds of the occultation echo observation. A full description of our error analysis is provided in the “Methods” section.

As previously mentioned, in additional contrast to other planetary BSR experiments, Dawn also has a relatively slow orbital velocity (~200 m s^−1^) with respect to Vesta’s surface. Since the relative motions of the target surface, the transmitter, and the receiver determine the Doppler shift that separates surface echoes from the frequency of directly transmitted radar waves, we therefore expect a small Doppler shift from Dawn’s BSR experiment at Vesta. As a result, a frequency resolution of a few hertz is necessary to resolve surface Doppler-shifted echoes from the direct signal in the received power-frequency spectra. To optimize the trade-off between signal-to-noise ratio (SNR), frequency drift, and spectral resolution, our final spectra are each averaged over two windows of 2.5-s integration time. Each spectrum is separated by a 1-s time interval in Fig. 2.

### Orbital BSR observations

Our analysis begins with the calculation of the expected differential Doppler shift between the direct signal and surface reflections. This is compared with the observed differential Doppler shift in the power spectra to confirm the detection of surface echoes. From the power spectrum of each echo, we measure radar cross-sections of Vesta’s surface and finally estimate the relative surface roughness of each echo site.

The differential Doppler shift *δf* between the direct signal and surface echo is attributed to the rotation of Vesta and the relative orbital motion of the Dawn spacecraft (see Supplementary Tables 2 and 3). Following the procedure for planetary BSR experiments^27^, theoretical *δf* is calculated for occultation entry of orbit 355. The surface echo is determined to have a Doppler-shifted frequency within ~2-20 Hz of that of the direct signal when considering uncertainties in (1) the ephemeris position of the spacecraft^28^; (2) the orbital velocity of the spacecraft due to deviations in the gravity field from the homogeneous model; (3) the precise latitude and longitude of the echo-site center, given the large area that is illuminated by the HGA beam at grazing incidence; and (4) the estimated radius (and subsequent rotational velocity) of the echo site, due to surface topography that is illuminated within the large radar footprint.

The theoretical value of *δf* ~2 Hz is therefore the lower limit of the differential Doppler shift between the surface echo and direct signal, under the assumptions that (1) the position and rotational velocity of the echo site on Vesta are well-represented by a point on the surface, and (2) Dawn’s orbital velocity is constant. Our calculation is consistent with the expectation of a small frequency separation due to Dawn’s low orbital velocity that is orders of magnitude smaller than observed in typical orbital BSR experiments at larger planetary bodies—such as for the orbital BSR experiment by the Mars Global Surveyor spacecraft, which detected radar reflections from the martian surface that were separated by as much as 10 kHz from the received direct signal^8^.

**Fig. 1:**
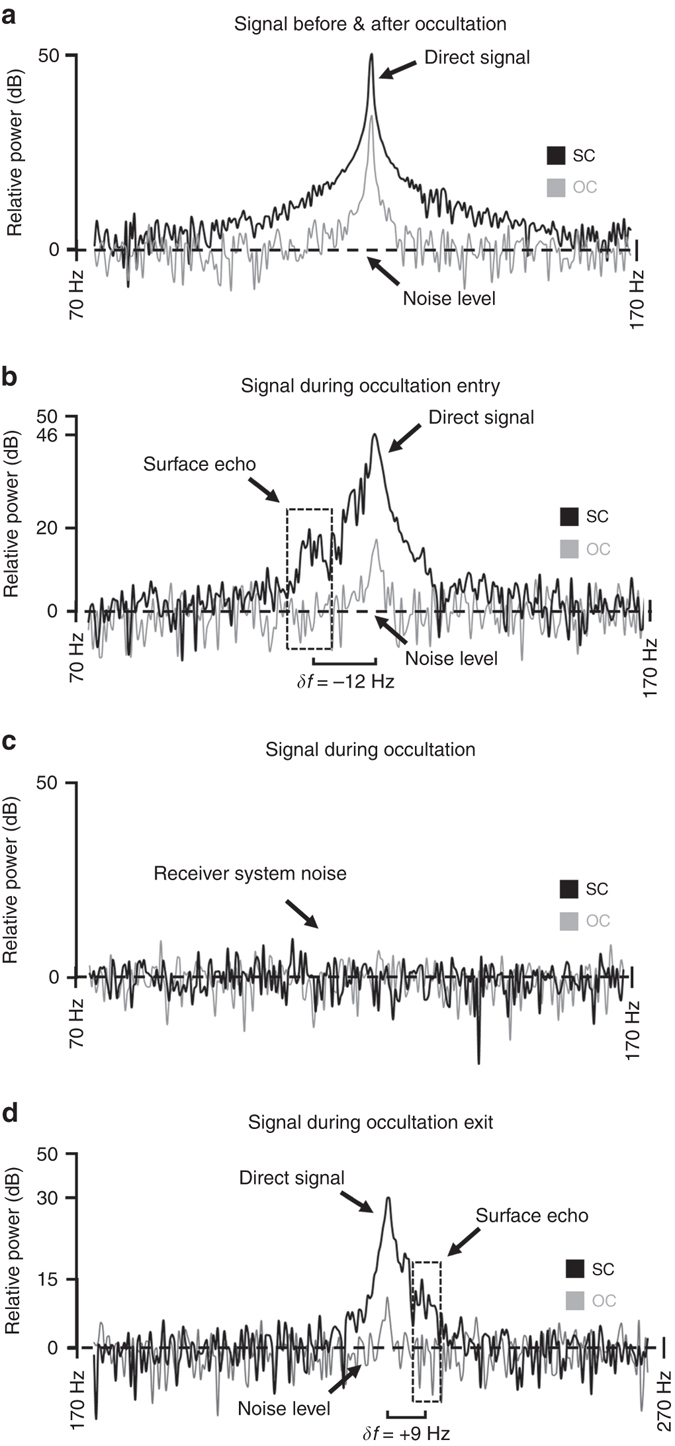
Typical progression of received radar signal over the course of an occultation. The frequency spectra show power received in the same circular (*SC*) and opposite circular (*OC*) polarization **a** before/after occultation, **b** during entry into occultation, **c** during occultation, and **d** during exit from occultation of orbit 355. All surface echoes during occultation entry are Doppler-shifted to lower frequencies than the direct signal, while all surface echoes during occultation exit exhibit Doppler shifts to higher frequencies than the direct signal

**Fig. 2:**
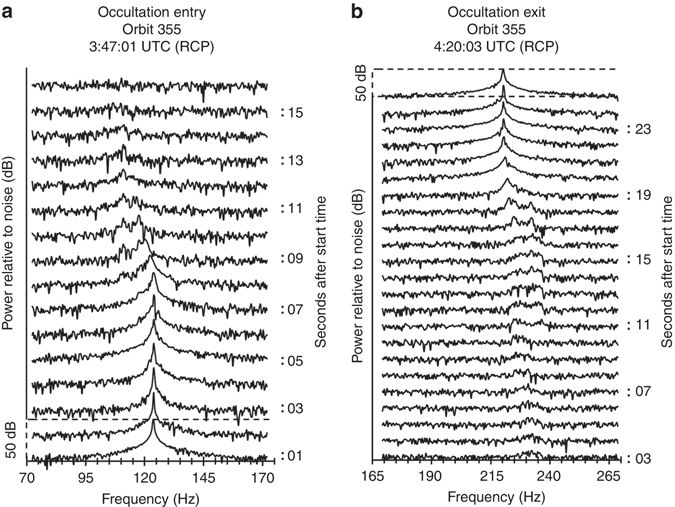
Progression of received radar signal throughout entrance into and exit from occultation during orbit 355. Occultation entry **a** spans ~16 s while occultation exit **b** spans ~25 s. Each spectrum is generated from two averages of 2.5-s integrated spectra, corresponding to a total of 5 s of radar data that start at each listed timestamp. Spectra are vertically offset for display purposes, where each successively higher spectrum corresponds to a step forward by 1 s

Figures 1 and 2 show the temporal progression of received power spectra from BSR observations during orbit 355. In Fig. 1, black spectra have the same circular polarization as the transmitted wave, RCP, while gray spectra correspond to the power received with opposite (left-hand) circular polarization, LCP.

Figure 1a shows the direct signal prior to occultation at its peak strength of 50 dB relative to the noise level. The presence of measurable LCP power in the direct signal indicates imperfection in the transmitting antenna and the high sensitivity of the 70-m DSN receivers, as the power in LCP is ~2.5% of (~16 dB below) the power measured in RCP. Panel (b) shows a typical power spectrum during Dawn’s entrance into occultation behind Vesta. Most of the direct signal is still visible to the receiver on Earth, while a secondary peak (the surface echo) emerges with a relative Doppler shift *δf* of −12 Hz with respect to the direct signal. The LCP component is now ~28 dB below the RCP peak, potentially because the main lobe of the antenna has become partially obstructed by Vesta’s surface.

Panel (c) shows typical RCP and LCP spectra observed amid full occultation of Dawn behind Vesta, and consist solely of receiver system noise. In the final panel (d), Dawn has partially exited from occultation behind Vesta. Most of the direct signal is again in the line of sight with the receiver, while a secondary surface echo peak is observed with a relative Doppler-shifted frequency +9 Hz higher than that of the direct signal. The LCP component is again ~28 dB weaker than the received RCP power, potentially due to partial obstruction of the antenna lobe.

The observed *δf* of −12 Hz during occultation entry is consistent with our calculated range of theoretical *δf* values between ~2 and 20 Hz, where the upper limit of theoretical *δf* is attributed to uncertainties in the precise position and velocity of the spacecraft, and in the location and subsequent radius and rotational velocity of each echo site. Furthermore, the direct signal has a frequency width of ~10 Hz at 31 dB below the peak at full strength, such that the secondary peak of an emerging surface echo is not observable until the direct signal has been sufficiently weakened behind Vesta or when *δf* is greater than half the frequency width of the diminishing direct signal.

This observation is further emphasized in Fig. 2a, which shows the progression of RCP power spectra over the course of 16 s during Dawn’s gradual entry into occultation behind Vesta during orbit 355. The lowest plotted spectrum is the same as that of panel (a) in Fig. 1, showing the direct signal prior to occultation. With each successive second (indicated by the next higher, vertically offset spectrum), direct signal power decreases as the HGA boresight passes behind Vesta’s horizon, while reflections from the surface emerge at a lower frequency until the topmost spectrum at which point the spacecraft is completely obscured by Vesta; only receiver noise is detected. Figure 2b shows the same temporal progression for occultation exit. The lowest spectrum shows the first moment of observable surface echo after occultation, and progresses to the top spectrum at which point the direct signal is fully in the line of sight with the receiver.

Another consequence of radar reflections at high grazing-incidence angle is that the polarization of the transmitted RCP waves are conserved in the forward-scatter direction (e.g., ref. ^25^). Since surface echoes do not contain a measurable LCP component, their spectra are excluded from Fig. 2. For reference, plots of the received null LCP power spectra are provided in Supplementary Fig. 3 during entry and exit from occultation of orbit 355.

**Fig. 3:**
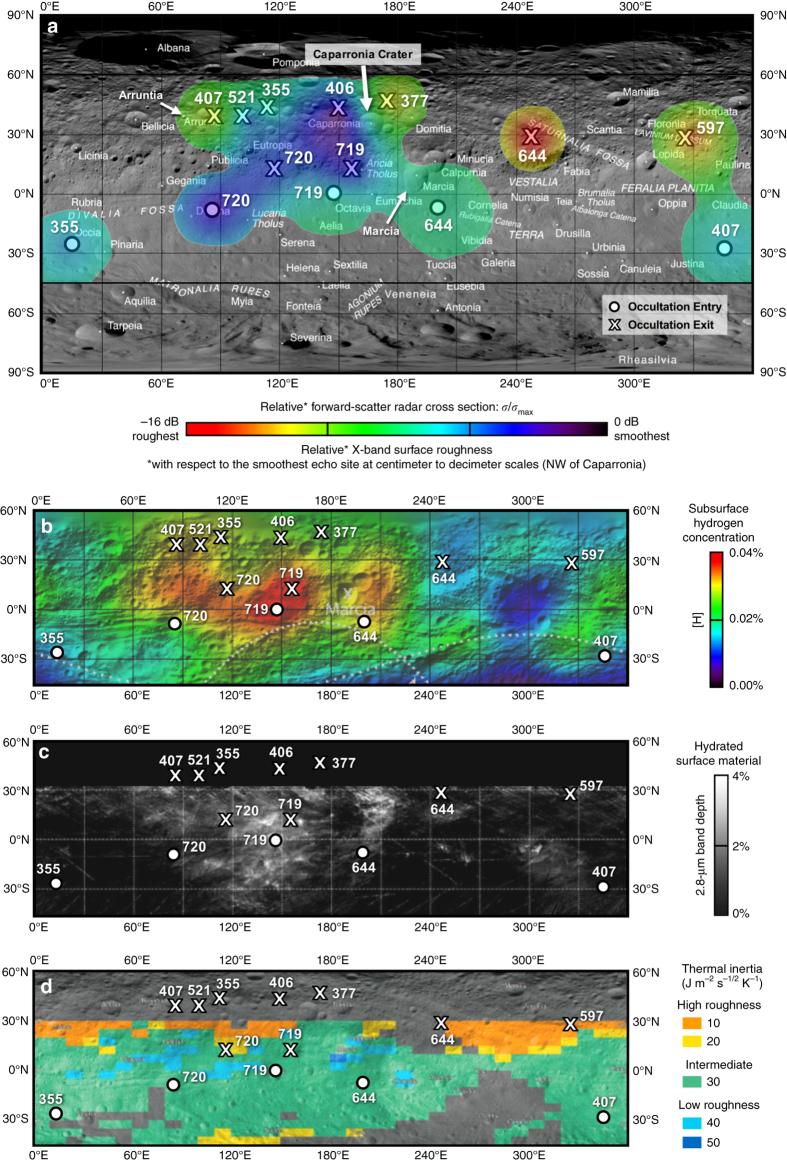
Comparison of BSR results with observations by the GRaND and VIR instruments aboard Dawn. Map **a** shows the distribution of relative radar cross section interpolated between echo sites and overlain upon an equirectangular projection of Vesta’s surface; **b** shows subsurface [H] to a depth of a few decimeters^17^; **c** shows the distribution of hydrated material at the surface^18^; and **d** shows the surface’s thermal inertia and multi-meter-scale topography modeled from VIR thermal observations^19^. O’s and X’s mark locations where BSR surface echoes have been detected during the associated orbit number

In total, 20 cases of surface echoes are detected at mid-latitudes, 14 of which (1) reflect from sites with minimal topographic variability, and (2) are sufficiently distinguishable from the direct signal to allow for characterization of the surface’s scattering properties in these regions. The radar-illuminated sites of the 14 echoes are plotted in Fig. 3a on an equirectangular projection of Vesta’s surface. High-resolution images of the smoothest and roughest observed echo sites are provided in Supplementary Fig. 5.

Vesta’s surface radar properties are explored hereafter using the forward-scatter radar cross section *σ* with units of km^2^. This parameter quantifies the cross-sectional surface area of a perfectly isotropic scatterer that would reflect the same echo power that is measured at Earth. For a given acquisition geometry, *σ* depends on the surface’s dielectric and roughness properties at the radar wavelength, and is determined from the ratio of received echo power to transmitted power^12^. Typically, *σ* is normalized to the surface area illuminated at each echo site (*σ* ^0^) and directly compared with backscatter measurements from other observations of the target surface (e.g., ref. ^29^) or of other planetary bodies. However, due to the ambiguity associated with topographic shadowing effects at grazing incidence and lack of directly comparable measurements in the forward-scattering regime, each *σ* measured at Vesta is instead assumed to have (1) approximately equal area illuminated during echo reflection, and (2) approximately equal power incident on the surface at 89°. Forward-scatter *σ* is then normalized to *σ*_max_, i.e., that of the site with maximum observed echo power. The resulting differences in relative forward-scatter radar cross sections (*σ/σ*_max_) are then used to infer variations in roughness at centimeter-to-decimeter scales across the surface of Vesta.

Figure 3a shows the resulting distribution of (*σ/σ*_max_) on Vesta, where the reference site for *σ*_max_ *= *3588 ± 200 km^2^ is located northwest of Caparronia crater (occultation exit of orbit 406). Values of (*σ/σ*_max_) range from –16.3 ± 0.5 dB at the site of weakest measured echo power, to zero dB at the *σ*_max_ reference site. The intrinsic reflective and absorptive (i.e., dielectric) properties of Vesta’s surface material, as estimated from Dawn’s VIR observations, are found to be constant throughout the upper regolith in X- and S-band^30^. For our sites which have minimum topographic variability, changes in (*σ/σ*_max_) are therefore attributed to spatial variations in surface roughness at centimeter-to-decimeter scales across Vesta. Radar echoes received from smoother sites reflect strongly in the forward direction and hence exhibit the strongest measured power in the regime of forward scatter, whereas weaker echoes are observed when reflected from rougher surfaces^12^. Echo sites with the highest (*σ/σ*_max_) ratios (*blue* in Fig. 3) therefore correspond to the smoothest observed surfaces on Vesta and sites with the lowest ratio (in *red*) represent the roughest observed surfaces. Table 1 contains measurements of (*σ/σ*_max_) for all surface echoes, including brief descriptions of the terrain associated with each reflection site^31^.

**Table 1:** Forward-scatter radar cross sections *σ* of Vesta’s surface measured at each echo site from high-incidence BSR surface reflections

Orbit no.	Occultation date & stage	Time at receiver HH:MM:SS (UTC)	Surface echo location (Lat., Lon.)	Terrain^31^	^a^ *σ* (km^2^)	^b^ *σ/σ*_max_ (dB)
355	24 DEC 2011 Entry	3:47:09	(25°S, 13°E)	Rheasilvia smooth terrain (rst)	1361 ± 19	–4.2 ± 0.2
355	24 DEC 2011 Exit	4:20:19	(43°N, 115°E)	Northern cratered trough terrain (nctt)	823 ± 16	–6.4 ± 0.3
377	28 DEC 2011 Exit	5:26:29	(46°N, 173°E)	nctt	262 ± 15	–11.4 ± 0.3
406	02 JAN 2012 Exit	13:11:48	(40°N, 149°E)	nctt	3588 ± 200	0(reference value^b^ for the site with maximum surface echo strength)
407	02 JAN 2012 Entry	17:06:36	(26°S, 346°E)	rst	1124 ± 17	–5.0 ± 0.3
407	02 JAN 2012 Exit	17:38:17	(39°N, 87°E)	Dark material near Arruntia crater	280 ± 12	–11.1 ± 0.3
521	23 JAN 2012 Exit	16:21:05	(38°N, 104°E)	nctt	1539 ± 27	–3.7 ± 0.3
597	06 FEB 2012 Exit	15:10:12	(29°N, 328°E)	Lavinium Dorsum	156 ± 13	–13.6 ± 0.4
644	15 FEB 2012 Entry	6:10:47	(7°S, 204°E)	Marcia crater ejecta	782 ± 21	–6.6 ± 0.3
644	15 FEB 2012 Exit	6:27:29	(29°N, 247°E)	Saturnalia Fossae	84 ± 8	–16.3 ± 0.5
719	29 FEB 2012 Entry	0:39:44	(0°N, 148°E)	Octavia crater	971 ± 87	–5.7 ± 0.5
719	29 FEB 2012 Exit	0:45:13	(13°N, 156°E)	Cratered highlands near Aricia Tholus	2369 ± 115	–1.8 ± 0.3
720	29 FEB 2012 Entry	4:59:51	(7°S, 86°E)	Divalia Fossae	3012 ± 43	–0.8 ± 0.2
720	29 FEB 2012 Exit	5:10:37	(13°N, 118°E)	Cratered highlands	2080 ± 42	–2.4 ± 0.3

^a^Smaller values of the forward-scatter radar cross section *σ* are attributed to weaker radar reflections from Vesta’s surface. Weaker radar reflections suggest rougher surfaces at the scale of centimeters to decimeters

^b^The largest radar cross section *σ*_max_ was measured from surface echoes located northwest of Caparronia crater, which is therefore the smoothest observed echo site at centimeter-to-decimeter scales. Decreasing values of (*σ/σ*_max_) are associated with progressively rougher surfaces at centimeter-to-decimeter scales

## Discussion

Vesta is presumed to have been largely depleted of volatiles during its differentiation^24^ but recent observations by Dawn’s GRaND and VIR instruments suggest the potential introduction of hydrated material through meteoritic impacts^17, 32^. Figure 3 shows the comparison of (a) the spatial distribution of relative forward-scatter radar cross section (*σ/σ*_max_)—inversely proportional to centimeter-to-decimeter-scale surface roughness—with (b) the distribution of [H] measured by GRaND at a depth of a few centimeters to decimeters^17^; (c) the distribution of hydrated surface material by VIR^18^; and (d) thermal inertia and multi-meter-scale topography modeled from VIR thermal observations^19^.

All echo sites within ±28° latitude from the equator have (*σ/σ*_max_) > –7 dB, [H] > 0.015% and overlap regions with hydrated surface material, suggesting that the smoothest observed terrains at centimeter and decimeter scales (relative to the smoothest reference site, which is observed northwest of Caparronia crater) are correlated with heightened [H] near Vesta’s equator. While [H], in turn, has been correlated with the presence of low-albedo surficial deposits of hydrated material (“dark material”)^17^, these deposits are proposed to have the mineralogical composition of carbonaceous chondrites^32^, which have indistinguishable dielectric properties from that of the surrounding lunar-like regolith—specifically, *ε′* is estimated to be ~2.4 for Vesta’s basaltic regolith^30^, *ε′* ~2.6 as measured for porous ordinary chondrites^33^, and *ε′* ~2.6–2.9 as measured for porous carbonaceous chondrites^34^. Hence, the observed correlation of radar reflectivity with [H] is due to textural variation and not in the dielectric properties of the surface.

The regolith should otherwise be particularly rough at the centimeter-to-decimeter scale in these geologic units—the cratered highlands, ejecta blankets of Octavia and Marcia crater, ejecta material from Rheasilvia, and Divalia Fossae^31^—in the absence of smoothing erosional processes, such as the melting, run-off and recrystallization of water ice after an impact, suggesting the potential presence of subsurface volatiles at these echo sites. The occurrence of heightened [H] with rougher surfaces, such as in the northern cratered trough terrain northeast of Caparronia crater potentially results from the following sequence: (1) initial smoothing due to impacts that induce ground-ice melting, run-off and re-crystallization of buried ice; and then (2) subsequent fracturing due to thermal erosion that is caused by the expansion and contraction of remnant volatile inclusions within the breccias and impactites that constitute a large fraction of the regolith’s surface and shallow subsurface (i.e., the first meter), due to significant diurnal temperature fluctuations. This hypothesized mechanism is further supported by the observation of gullies and flow features on crater walls from Dawn Framing Camera (FC) images that suggest transitional melting of ground-ice during the post-impact process^35^. Above 30°N, roughness is observed to lessen with increasing [H], suggesting less fracturing occurring from thermal erosion at higher latitudes, which is consistent with minimal solar illumination at northern latitudes due to seasonal shadowing during Dawn’s orbit of Vesta (e.g., ref. ^19^).

On the Moon, there is a strong correlation between the surface age of geologic units and their radar backscatter properties, implying that impact cratering is the dominant process that governs the texture of the lunar regolith at centimeter-to-decimeter scales^36^. Younger units, especially crater ejecta, are rough at centimeter and decimeter scales due to the presence of shocked and fractured meter- and centimeter-sized fragments from impacts; over time, older cratered surfaces are covered with a layer of fine debris that buries rock fragments, therefore appearing smooth at the surface to radar at centimeter and decimeter scales^36^. However, we do not observe the same correlation on Vesta between relative surface roughness and relative surface ages. Williams et al.^13^ studied the chronology of Vesta’s various geologic units through crater counting methods and developed two models for surfaces ages: one extrapolated from lunar-derived chronology, and the other from models of asteroid belt dynamics. In Supplementary Fig. 4 we plot the approximate surface age of each echo site’s geologic unit for each chronology against their observed relative radar cross section (*σ/σ*_max_) values, but we find no observable correlation of radar scattering properties with surface age, suggesting that cratering cannot be the only process shaping Vesta’s surface texture.

The lack of observable correlation between cratering and radar-wavelength surface texture is further evidenced by high-resolution Dawn FC images of Vesta at the sites of strongest and weakest BSR surface-echo reflections—i.e., the smoothest and roughest sites observed by BSR, respectively, at centimeter-to-decimeter scales. Panel (a) of Supplementary Fig. 5 shows a regional view of the smoothest echo site at 66 m per pixel resolution on the left, and a subset of the echo site at 18 m per pixel resolution on the right. Panel (b) shows the roughest echo site at 62 m per pixel and 22 m per pixel. We do not observe a correlation between surface topography at the scale of meters to tens of meters with the centimeter-to-decimeter-scale surface roughness that is observed by Dawn’s BSR investigation.

Together, these observations suggest that unlike the Moon, impact cratering processes cannot solely explain Vesta’s surface roughness, and that fracturing arising from subsurface volatile occurrence may have contributed to the formation of the current surface texture. This is further supported by the thermal inertia map of Vesta’s surface in Fig. 3d, which also shows no correlation between BSR-derived centimeter-to-decimeter-scale roughness and the multi-meter-scale topography that is derived during the process of thermal inertia modeling^19^.

In total, we have identified 10 probable sites for potential shallow subsurface volatile occurrence that include occultation entry during orbits 644, 719, and 720, and occultation exit during orbits 355, 406, 407, 521, 719, and 720. Each corresponds to sites with the smoothest to intermediate surface roughness on Vesta and exhibit heightened subsurface [H] between 0.025 and 0.04% as observed by GRaND^17^.

With regard to future landing and sample collection missions on asteroids^20^, the observed variation of centimeter-to-decimeter-scale surface roughness across Vesta further emphasizes the importance of these opportunistic observations to be systematically carried out to support safe landing, proper anchoring, and optimized collection of potentially volatile-enriched samples. BSR observations constrain the spatial variability of surface roughness on small bodies and can thereby support safe trafficability—especially for equatorial regions as are observed on Vesta, which are frequently considered for landing and sampling return sites^20^. While topographic maps derived from orbital observations provide first-order information about large-scale obstacles, high-resolution orbital BSR observations yield information about surface roughness at centimeter-to-decimeter scales that cannot be derived from Earth-based observations. On Vesta, no correlation is observed between topographic elevation and the distribution of surface roughness.

In summary, the orbital BSR experiment at asteroid Vesta emphasizes the importance of utilizing standard communications antennas aboard spacecraft to derive constraints on surface roughness at sub-topographic scales. In our future work, we will apply the same analysis to BSR observations of icy asteroid Ceres, the second target of the Dawn mission to understand potential subsurface volatile occurrence.

## Methods

### Experimental constraints and data selection

During the BSR experiment at Vesta, the Dawn spacecraft’s HGA was used to transmit telemetry data at X-band radar frequency (8.435 GHz, 3.55 cm wavelength) in RCP, while the three 70-m DSN antennas on Earth—at Goldstone (USA), Canberra (Australia) or Madrid (Spain)—were used to receive^22^. The three receiving systems have the same measurement requirements in terms of noise temperature, antenna gain, pointing loss, and polarization loss as listed in Supplementary Table 1. In two-way coherent downlink mode, Dawn’s transmission frequency is driven by the DSN’s uplink frequency and yields a Doppler stability (Δ*f/f*) of 10^−12^ over 60-s measurements, i.e., a frequency drift of ~0.0001 Hz s^−1^ ^1^. In one-way or non-coherent downlink mode—used when the uplink signal is expected to be unavailable, such as the minutes preceding and following an occultation—the transmission frequency is driven by an onboard internal auxiliary oscillator with a maximum frequency drift of 0.05 Hz s^−1^ ^37^. One-way downlink mode contains too much Doppler noise to be used for gravity science^23^ but is sufficiently stable for BSR measurements since they are integrated over a much shorter timespan—a few seconds, as opposed to one minute for gravity science measurements—and because we are measuring the relative Doppler shift between the surface echo and direct signal, which are equally affected by slow Doppler changes.

Dawn’s HGA was almost constantly pointed at ground stations on Earth for communication^1^, so by continuously transmitting basic telemetry information, we had the opportunity to observe surface reflections of the radar signal just before and after each occultation of the Dawn spacecraft behind Vesta at highly oblique incident angles of ~89° as depicted in Supplementary Fig. 1, similar to geometry of the BSR experiment at Mars by the Mars Global Surveyor^38^. In order to analyze the resulting BSR data, we selected occultations that occurred specifically during Dawn’s lowest-altitude mapping orbit (LAMO) around Vesta, and using the HGA to ensure the strongest observable surface echoes. During LAMO, there were 16 unique orbits during which an occultation occurred, and therefore 16 individual entries and 16 individual exits.

Out of these events, several were discarded based on the following criteria: (1) if the radar amplitude data files containing RCP and LCP were copies of each other; (2) if the direct (carrier) signal’s power did not consistently exhibit 50 dB strength before or after its occultation; (3) if the surface echo was indistinguishable from the noise (i.e., < ~3 dB); (4) if the window of occultation entry or exit was so brief that the signal disappeared faster than our temporal resolution (e.g., if the carrier dropped by 30 dB within a few seconds, and usually showed no indication of a measurable surface echo); (5) if the *δf* was too small to distinguish the surface echo’s power from that of the carrier; or (6) if the surface echo occurs in a region of high topographic variability with respect to the incident HGA beam size, which is assessed below. In all, five entries and nine exits passed our criteria for analysis and measurement of *σ* (km^2^) and *σ/σ*_max_ (dB). The following sections describe the method used to process and analyze the resulting BSR data. Supplementary Table 1 summarizes the acquisition parameters of the BSR experiment.

### Processing DSN BSR data

Radio waves received by the DSN 70-meter antennas are recorded as amplitude (voltage) versus time, and are collected in two channels: RCP and LCP. Within each channel, amplitude data is recorded in two components, in-phase *I* and quadrature *Q*, which correspond to the real and imaginary parts of the complex voltage, respectively.

The raw modulated telemetry data collected at the DSN antenna is sampled at a rate of 16 kHz. In addition, a Doppler shift correction is applied to the X-band receiving frequency in order to counteract the calculated Doppler shift induced by Dawn’s orbit around Vesta. However, the DSN utilizes a local oscillator to subtract the ephemeride Doppler shifting, and what persists is a low frequency component of error, which we observe is as much as 300 Hz. The offset frequency of the carrier signal is observed to decrease as the spacecraft moves further away from Earth during occultation entry—and as expected, increases as the spacecraft moves closer toward Earth after leaving occultation. Given that most of the BSR observations of occultations were conducted in one-way mode, the accuracy of the predicted receiving frequency was also diminished in the time leading up to and following these unique orbital geometries. Hence, when radar amplitude data is plotted against time, a change is observed in the overall envelope frequency of the sinusoidal amplitudes over the course of, for example, 1 min, during which the carrier signal may shift from 200 to 100 Hz. Notably, this frequency offset does not impact the relative Doppler shift between the surface echo and direct signal.

To seek surface echoes, the DSN *I*-and-*Q* amplitude time series data is converted into the frequency domain by taking the complex fast Fourier transform. The power frequency spectrum is generated in voltage-squared per Hz such that:

1 $${P_{{\rm{spec}}}} = \frac{1}{{{t_{{\rm{spec}}}}}}{\left| {{\rm{FFT}}\left( {{A_I} + i{A_Q}} \right)} \right|^2}$$

The duration *t*_spec_ over which to generate each power spectrum is selected only after assessing the theoretical Doppler separation *δf* between the direct signal and any surface echoes, as this dictates the frequency resolution necessary to accurately distinguish each surface echo. Background noise is smoothed by averaging power spectra together, but surface-echo strength may be diminished in the process. The latter occurs when spectra are averaged over times that lack a surface-echo signal or while the echo has changed in frequency. The final parameters used to generate power spectra are outlined at the end of the following section.

### Calculating the differential Doppler shift

In order to verify the detection of surface reflections within BSR power spectra, the theoretical differential Doppler shift *δf* is calculated between the direct signal and grazing surface-reflected echoes, and compared with measured values. Theoretical *δf* is estimated from the known positions and line-of-sight velocities between (1) the Dawn spacecraft (D) at the time of transmission; (2) the center coordinate of the radar-illuminated surface on Vesta (Vpt) when the signal reaches the surface; and (3) the receiving antenna on Earth (E) after accounting for light travel time; each of which are extracted from the reconstructed trajectory of Dawn’s orbit that is provided in SPICE ephemeride data^28^.

For occultation entry during orbit 355, Supplementary Fig. 2 shows the instantaneous total velocities *v* of Dawn, the surface point of reflection on Vesta (hereafter referred to as “the echo site” or “the site”), and the receiving antenna on Earth within the bistatic plane—defined as the plane containing all three bodies at a given moment^12^. All positions and velocities of D, Vpt and E are obtained with respect to Vesta’s inertial frame of reference, such that the origin of the Cartesian coordinate system is Vesta’s center of gravity and Vesta’s equator defines the *xy*-plane. The individual components of each body’s instantaneous velocity is listed in Supplementary Table 2 for occultation entry during orbit 355, where each velocity *v*_A_ is given in m s^-1^ along the line of sight with respect to the position ${\hat r_{\rm{B}}}$. Velocity components are defined to be positive if body A is moving toward target B, and negative if away. For example, the notation ${\it v_{\rm{D}}}{\hat r_{{\rm{Vp\it t}}}}$ = −82.2 m s^−1^ (column 2, row 4) indicates that Dawn is traveling away from the echo site at a speed of 82.2 m s^−1^.

The differential Doppler shift *δf* between the surface echo and direct signal is calculated by:

2 $$\delta f = \Delta {f_{{\rm{echo}}}} - \Delta {f_{{\rm{direct}}}}$$

where the absolute Doppler shift Δ*f* of the direct signal or surface echo depends on the relative velocity of the transmitting and receiving bodies along their line of sight as described by Simpson^27^. Supplementary Table 3 shows the mathematical definition and calculation of individual contributions to the total theoretical differential Doppler shift (*δf*_total_) between the surface echo and direct signal, which are the result of motions along the line of sight between Dawn and Earth (column 1), Dawn’s orbital motion around Vesta (column 2), and Vesta’s rotation (column 3).

The absolute Doppler shift of the direct signal Δ*f*_direct_ is first calculated from the combination of Dawn’s instantaneous line-of-sight velocity toward the receiving antenna on Earth (${\it v_{\rm{D}}}{\hat r_{\rm{E}}}$), and Earth’s line-of-sight velocity toward Dawn’s position (${v_{\rm{E}}}{\hat r_{\rm{D}}}$). The differential Doppler shift due to Dawn’s orbital motion (*δf*_orbit_) is then calculated from the Doppler shift contributed by ${v_{\rm{D}}}{\hat r_{{\rm{Vp\it t}}}}$ and its difference from Δ*f*_direct_. In turn, the differential Doppler shift contributed by the rotation of Vpt (*δf*_rotation_) on Vesta’s surface is calculated from the Doppler shift contributed by ${v_{{\rm{Vp\it t}}}}{\hat r_{\rm{D}}}$ and ${v_{{\rm{Vp\it t}}}}{\hat r_{\rm{E}}}$ and their difference from Δ*f*_direct_. The combined Doppler shift contributions of *δf*_orbit_ and *δf*_rotation_ yield the total theoretical *δf*_total_, which is calculated to range from ~2 Hz, as listed in Supplementary Table 3, to as much as 20 Hz when considering uncertainties in spacecraft position and Vesta’s rotational velocity (detailed further below in our error analysis). Hence, the surface echo during occultation entry of orbit 355 is calculated to have a frequency shift that ranges from ~2 to 20 Hz higher or lower than that of the received direct signal.

This calculation confirms that in the configuration of grazing incidence during occultation observations of Vesta by Dawn, and due to the spacecraft’s low orbital velocity of 200 m s^−1^, the frequency separation *δf* between surface echoes and the direct signal will be small. Higher frequency spectral resolution is therefore necessary to distinguish the Doppler-shifted surface echoes from the direct signal at Vesta. However, this requires longer integration time of the observation. We chose 2.5-s integration time to obtain a frequency spectral resolution of ~0.4 Hz as a tradeoff between SNR, frequency drift and resolution. Our final frequency spectral analysis averages two 2.5-s looks, and repeats this calculation shifting the start time of the averaging by 1 s. The resulting spectra are exemplified for occultation entry and exit during orbit 355 in Supplementary Fig. 3, which shows the power received in both RCP (reproduced from Fig. 2) and in LCP.

### Calculating the radar cross section at high incidence

In order to assess the surface’s geophysical properties that contribute to the observed surface echoes, the radar cross section *σ* of Vesta’s surface is calculated in m^2^ from the BSR equation^12^. This parameter is defined as the effective surface area that isotropically scatters the same amount of power as the echo site on Vesta, such that larger values of *σ* are associated with stronger surface echoes. Assuming (1) each echo site has approximately the same surface area illuminated by radar and (2) are observed at the same geometry (89° incidence), relative differences in *σ* imply differences in geophysical properties of the surface.

The latter assumption is supported by excluding surface echoes that occurred in regions of high topographic variability with respect to the incident HGA beam diameter, where the illuminated surface area is estimated using a first-order spherical approximation to Vesta’s surface. Notably, this approximation excludes the effects of shadowing and diffraction, which are difficult to quantify at grazing incidence, and does not account for deviations of Vesta’s shape from the sphere within the large area illuminated by the HGA beam. Our first-order estimation yields an elongated radar footprint of ~51 km along the line of sight between Dawn’s HGA and Earth’s receiving antenna, and ~11 km in diameter perpendicular to the line-of-sight. Topographic variability is assessed by calculating the root-mean-square height *h*_rms_ using elevations from Vesta’s digital terrain model^39^ within an 11.5° by 11.5° grid (~51 × 51 km) centered on each echo site’s coordinates, and must be sufficiently smaller than the incident 11-km beam diameter. Calculated *h*_rms_ range from 0.002 to 0.13 km. All but one echo site had *h*_rms_ < 0.11 km (i.e., topographic variability of 1% of the incident beam diameter). The echo site of occultation entry during orbit 377 exceeded this criteria and is therefore excluded from the following analyses.

Since HGA transmissions are measured continuously throughout the minutes that precede and follow an occultation of the spacecraft behind Vesta, only engineering data is included in the raw telemetry, and DSN receiver calibration measurements are not included with the raw radar data set. Because the BSR data are not calibrated in absolute voltage, calculating *σ* therefore requires calibrating the measured power to a known reference. This is made possible by calculating the theoretical received power of the direct signal *P*_r dir|calc_ in watts, and comparing with measured received power *P*_r dir|meas_ in data units. *P*_r dir|calc_ is calculated from the one-way radar equation and depends on the transmitted power *P*_t_, gain of the transmitting *G*_t_ and receiving *G*_r_ antennas, the distance *R*_DE_ between the transmitter aboard Dawn and the receiving antenna on Earth, and summed losses *L*. The one-way radar equation for *P*_r dir|calc_ (W) is then as follows^12^:

3 $${P} {_{{\rm{r}} \,{\rm{dir|calc}}}} = \frac{{{P_{\rm{t}}}{G_{\rm{t}}}{G_{\rm{r}}}{\lambda ^2}}}{{{{\left( {4\pi {R_{{\rm{DE}}}}} \right)}^2}L}}$$

where the nominal range of each parameter—except for time-dependent *R*_DE_—is provided in Supplementary Table 1. Note that losses contributed by the DSN 70-m antennas are published in the telecommunications parameters of the Deep Space 1 mission^40^.

*P*_r dir|meas_ is evaluated by measuring the area under the curve in non-logarithmic units during a time when the direct signal is not obstructed by Vesta. Since the data is discrete, *P*_r dir|meas_ is the sum of the power in each frequency bin multiplied by the width of each frequency bin (subtracted by the noise power in the same bandwidth):

4 $${P_{{\rm{r}}\,{\rm{dir|meas}}}} = \left( {{\sum} {{P_{\rm{i}}} \cdot \Delta {f_{{\rm{step}}}}} } \right) - \overline {{P_{\rm{N}}}} \cdot {f_{{\rm{BW}}}}$$

where frequency step Δ*f*_step_ is the spectral resolution of ~0.4 Hz, as previously determined when calculating the differential Doppler shift; *P*_i_ is the non-logarithmic power in data units of each discrete point measured within a 10-Hz bandwidth (*f*_BW_) of the direct signal peak; and $\overline {{P_{\rm{N}}}}$ is the average noise power (data units Hz^−1^) in the spectrum. The conversion factor between watts and power measured from BSR data units is therefore:

5 $${C_{{\rm{ToWatts}}}} = \frac{{{P_{{\rm{r}}\,{\rm{dir}}\left| {{\rm{calc}}} \right.}}\left[ {{\rm{Watts}}} \right]}}{{{P_{{\rm{r}}\,{\rm{dir}}\left| {{\rm{meas}}} \right.}}\left[ {{\rm{Data}}\,{\rm{Units}}} \right]}}$$

and the BSR equation, solved for *σ*, is then:

6 $${\sigma _{\left( {{{\rm{m}}^{\rm{2}}}} \right)}} = \frac{{{{\left( {4\pi } \right)}^3}{R_{\rm{t}}}^2{R_{\rm{r}}}^2L}}{{{G_{\rm{t}}}{G_{\rm{r}}}{\lambda ^2}}}\left( {\frac{{{P_{{\rm{r}}\,{\rm{echo}}|{\rm{meas}}}}}}{{\left( {1 - {X_{{{\rm{P}}_{\rm{t}}}}}} \right){P_{\rm{t}}}}}} \right)$$

where *X*_Pt_ is the fraction of incident power that has been reduced due to partial obstruction of the HGA beam by Vesta’s surface, and *P*_r echo|meas_(W) =*P*_r echo|meas_(Data Units) × *C*_ToWatts_. By measuring the received direct signal *P*_r dir|meas_ at a time close to each measured surface echo—10 s before a given occultation entry, and 10 s after an occultation exit—we minimize variations in the transmitting and receiving system characteristics, including changes in the HGA’s pointing accuracy, and potential differences in DSN receiver losses due to the use of different receiving stations with different system temperatures and atmospheric conditions. Hence, the measurement of the radar cross section *σ* (m^2^) becomes independent of *P*_t_, *G*_t_, *G*_r_, *L* and *λ*, such that:

7 $${\sigma _{\left( {{{\rm{m}}^{\rm{2}}}} \right)}} = \frac{{{{\left( {4\pi } \right)}^3}R_{{\rm{DVpt}}}^2R_{{\rm{EVpt}}}^2}}{{\left( {1 - {X_{{{\rm{P}}_{\rm{t}}}}}} \right)R_{{\rm{DE}}}^2}}\left( {\frac{{{P_{{\rm{r}}\,{\rm{echo|meas}}}}}}{{{P_{{\rm{r}}\,{\rm{dir|meas}}}}}}} \right)$$

where *R*_DVpt_ is the distance between the transmitter aboard Dawn and the echo site on Vesta’s surface at the time of the observed surface echo; *R*_EVpt_ is the distance between the receiving antenna on Earth and the echo site at the time of the observed surface echo; and *R*_DE_ is the distance between Dawn’s HGA and the receiver on Earth at the time when *P*_r dir|meas_ is measured (10 s before occultation entries, and 10 s after occultation exits).

Typically, the radar cross section is then normalized to the areal extent illuminated by the radar (*σ* ^0^), and is reported in regime of diffuse backscatter due to the use of Earth-based radar antennas as both transmitter and receiver for many observations (e.g., ref. ^29^)—where at increasingly high angles of incidence, the diffuse component of radar backscatter dominates the received signal^14^. BSR observations at Vesta are conducted in the forward scatter regime, however, whereby radar waves predominantly scatter in the forward direction and almost entirely within the plane of incidence^12^. In this regime, the polarization of a circularly transmitted wave is also conserved in major part even after reflection from the target’s surface^25^. Dawn’s measurements of *σ* ^0^ on Vesta’s surface are therefore not directly comparable with those observed in the backscatter regime on other planetary bodies.

While the lack of comparability might be overcome by deriving surface roughness from *σ* ^0^, two sources of uncertainty remain: (1) the absolute surface area contributing to forward-scattered surface echoes is difficult to quantify due to the effects of shadowing and multiple scattering that become important at such high, grazing incidence^38^; and (2) there is no appropriate scattering model to address the impact of wavelength-scale surface roughness on radar reflections at grazing angles of incidence approaching 90°^41^.

Instead, we calculate relative *σ* across Vesta’s surface with respect to the strongest observed surface reflection *σ*_max_ by measuring *σ* when the direct signal is ~25 dB above the noise level for all surface echoes, and employ the assumption that the illuminated surface area is approximately equal for each site. Since incident power is also assumed equal for all surface echoes (and therefore *X*_Pt_ assumed constant for Equations 6 and 7), we estimate that at least 50% of the HGA beam is obscured behind Vesta’s surface (*X*_Pt_ = 0.5) and report *σ* (km^2^) as a lower limit for each echo site.

Under the above assumptions of equal incident power and equal surface area illuminated at 89° incidence, the relative strengths of surface echo reflections (*σ/σ*_max_) can then be attributed to differences in the relative reflectivity of the surface material itself or variations in the roughness of the surface at the scale of the radar wavelength^14, 26^. Potentially greater obstruction of the incident power would result in an increase of *σ* (km^2^), but assuming equal obstruction for all surface echoes, this does not change the relative radar cross section (*σ/σ*_max_).

### Uncertainty in the differential Doppler shift

The primary sources of uncertainty in theoretical *δf* include the positions and velocities of (1) the spacecraft and (2) the radar-illuminated surface echo site on Vesta as listed in Supplementary Table 4. We assume that uncertainty in the position and velocity of Earth is negligible at such distances. For a given parameter *Y* ± Δ*Y* that depends on multiple variables *X*_i_ ± (Δ*X*)_i_, we calculate Δ*Y* by summing in quadrature the partial derivative of each contributing variable (*∂X*_i_ */∂Y*) multiplied by its uncertainty (Δ*X*)_i_.

The position of the Dawn spacecraft is provided in Cartesian coordinates from SPICE ephemerides with an uncertainty of ± 3 m in the radial, along-track and cross-track directions from the reconstructed trajectory of LAMO^28^, while the error in the position of the echo site on Vesta’s surface is calculated from (1) uncertainty in the radius of the echo site from Vesta’s center ± ~0.5 km—due to topography within the large surface area illuminated by the HGA beam at grazing incidence—and (2) uncertainty in the latitude and longitude of the echo site center by ± ~0.2°. Uncertainties in the geodetic coordinates of the echo site are then converted to Cartesian coordinates at a height above or below a reference triaxial ellipsoid—which is defined using the best-fit ellipsoid derived from Hubble light-curve observations of Vesta, where *R*_x_ = 289 km, *R*_y_ = 280 km and *R*_z_ = 229 km^28, 42^. Uncertainties in the *x*, *y* and *z* coordinates of the echo site for occultation entry of orbit 355 are on the order of ~0.7 km for the occultation entry of orbit 355.

With regard to spacecraft velocity, the SPICE ephemerides containing Dawn’s state vector were released by the optical navigation team in 2012^28^ before the peer-reviewed publication of Vesta’s gravitational solution in 2014^11^. Hence, Dawn’s reconstructed trajectory does not include variations in orbital velocity due to the heterogeneous gravity field that exhibits accelerations between −1000 mGal and +2000 mGal (−1 cm s^−2^ and +2 cm s^−2^) relative to the homogeneous model^11^. Since each frequency spectrum produced in our BSR analysis is averaged over 5-s observations, unpredicted gravitational accelerations contribute –5 to +10 cm s^−1^ uncertainty in Dawn’s velocity vector.

The rotational velocity of the echo site on Vesta’s surface depends on the distance of the site from Vesta’s center and the rotation period, the latter of which is known to high precision^11^. Given the large extent of surface area illuminated by the HGA beam on Vesta, we use the radius at the center of the echo site to calculate a representative rotational velocity. During occultation entry of orbit 355, uncertainty of ± 0.5 km in the radius yields an uncertainty of ± 16 cm s^−1^ in the echo site’s rotational velocity.

Using the above-derived uncertainties in the positions and velocities of each body, we calculate the propagation of error into each line-of-sight velocity between Dawn, the surface echo site on Vesta, and the antenna on Earth that contribute to the calculation of theoretical *δf*. Uncertainty in the velocity of body A projected along the line of site with body B (${\it v_{\rm{A}}}{\hat r_{\rm{B}}}$) is calculated from the propagation of error in (1) the velocity vector of A, (2) the position of A, and (3) the position of B. Hence, the theoretical differential Doppler shift is calculated to range between ~2 and 20 Hz, and is consistent the observed *δf* of –12 Hz (Fig. 1b) for the surface echo measured during orbit 355, occultation entry.

### Uncertainty in the absolute and relative radar cross section

The primary sources of uncertainty in the radar cross section include (1) HGA pointing error, (2) uncertainty in the measurement of received power, (3) uncertainty in the position of the Dawn spacecraft, and (4) uncertainty in the position of the echo site center—where the latter two uncertainties have been previously quantified above.

When outside of occultation, the measured power received from the direct signal *P*_r dir|meas_ varies as a result of HGA antenna pointing inaccuracy due to unpredicted uneven gravitational torques on the spacecraft’s large solar panels while in Vesta’s microgravity environment^43^. The spacecraft’s reaction wheels are used to counteract accumulated spacecraft pointing errors, but one of the four wheels failed prior to Dawn’s arrival at Vesta^43^. Furthermore, corrections only occurred once every 1-3 days during LAMO, such that antenna pointing error increased steadily from zero to ~0.4° between corrections, and even exceeded 1.0° on a few occasions^43^. To quantify fluctuations in the direct signal power on the order of tens of seconds near the time of each surface echo observation, we measure the variation of *P*_r dir|meas_ over 30 s preceding an occultation entry (and over 30 s following an occultation exit). We find that *P*_r dir|meas_ varies less than ±2% for 11 of the 14 occultation observations but as much as ±8% before occultation entry of orbit 719.

The standard error in the measurement of *P*_r echo|meas_ and *P*_r dir|meas_ are quantified by deviations of noise power from the mean. We compute the standard deviation of noise in a given spectrum over frequencies where no signal is present (from −6 to −1 kHz and from 1 to 6 kHz), and find that the standard deviation of a given spectrum ranges from ~0.14 $\overline {{P_{\rm{N}}}}$ to 0.17 $\overline {{P_{\rm{N}}}}$. We use the upper limit of 0.17 $\overline {{P_{\rm{N}}}}$ as a conservative estimate for all uncertainties in power measurement.

Together, the above errors in HGA antenna pointing, measurement of received power, spacecraft position and echo site position amount to uncertainties in *σ* (km^2^) that range from 1% to 10% depending on the surface echo—see Table 1. Subsequent error in the relative radar cross section (*σ/σ*_max_) range from zero dB for the strongest surface echo reflection to ± 0.5 dB for the weakest surface echo reflection.

### Data availability

Raw telemetry data from Dawn’s orbital BSR experiment at Vesta were generated from receiver output at stations of the NASA DSN and are managed by the NASA Jet Propulsion Laboratory of the California Institute of Technology. The unprocessed time-domain BSR amplitude data used in this study are available upon request from the corresponding author.

## Electronic supplementary material

Supplementary Information

## References

[1] Thomas, V. C. & Makowski, J. M. in *The Dawn Mission to Minor Planets 4 Vesta and 1 Ceres* (eds Russell, C. T. & Raymond, C. A.) 175-249 (Springer, 2012).

[2] Butler BJ, Muhleman DO, Slade MA (1993). Mercury: full-disk radar images and the detection and stability of ice at the north pole. J. Geophys. Res..

[3] Simpson RA, Tyler GL, Häusler B, Mattei R, Pätzold M (2009). Venus Express bistatic radar: high-elevation anomalous reflectivity. J. Geophys. Res..

[4] Tyler GL, Eshleman VR, Fjeldbo G, Howard HT, Peterson AM (1967). Bistatic-radar detection of lunar scattering centers with Lunar Orbiter I. Science.

[5] Nozette S (1996). The Clementine bistatic radar experiment. Science.

[6] Patterson GW (2017). Bistatic radar observations of the Moon using Mini-RF on LRO and the Arecibo Observatory. Icarus.

[7] Fjeldbo G, Kliore A, Seidel B (1972). Bistatic radar measurements of the surface of Mars with Mariner 1969. Icarus.

[8] Simpson RA, Tyler GL (2001). Mars Global Surveyor bistatic radar probing of the MPL/DS2 target area. Icarus.

[9] Pérez-Ayúcar M, Lorenz RD, Floury N, Prieto-Cerdeira R, Lebreton J-P (2006). Bistatic observations of Titan’s surface with the Huygens probe radio signal. J. Geophys. Res..

[10] Pätzold M (2007). Rosetta radio science investigations (RSI). Space Sci. Rev..

[11] Konopliv AS (2014). The Vesta gravity field, spin pole and rotation period, landmark positions, and ephemeris from the Dawn tracking and optical data. Icarus.

[12] Willis, N. J. & Griffiths, H. D. *Advances in Bistatic Radar* (SciTech Publishing, 2007).

[13] Williams DA (2014). The chronostratigraphy of protoplanet Vesta. Icarus.

[14] Thompson TW, Ustinov EA, Heggy E (2011). Modeling radar scattering from icy lunar regoliths at 13 cm and 4 cm wavelengths. J. Geophys. Res..

[15] Campbell BA (2010). Earth-based 12.6-cm wavelength radar mapping of the Moon: New views of impact melt distribution and mare physical properties. Icarus.

[16] Shepard MK (2001). The roughness of natural terrain: a planetary and remote sensing perspective. J. Geophys. Res..

[17] Prettyman TH (2012). Elemental mapping by Dawn reveals exogenic H in Vesta’s regolith. Science.

[18] De Sanctis MC (2012). Detection of widespread hydrated materials on Vesta by the VIR imaging spectrometer on board the Dawn mission. Astrophys. J. Lett..

[19] Capria MT (2014). Vesta surface thermal properties map. Geophys. Res. Lett..

[20] ElShafie A, Heggy E (2013). Dielectric and hardness measurements of planetary analog rocks in support of in-situ subsurface sampling. Planet. Space. Sci..

[21] Grasset O (2013). JUpiter ICy moons Explorer (JUICE): An ESA mission to orbit Ganymede and to characterise the Jupiter system. Planet. Space. Sci..

[22] Taylor, J. Dawn telecommunications. *NASA DESCANSO Design and Performance Summary Series* Article **13**, (2009).

[23] Konopliv, A. S. et al. in *The Dawn Mission to Minor Planets 4 Vesta and 1 Ceres* (eds Russell, C. T. & Raymond, C. A.) 465-466 (Springer, 2012).

[24] Russell, C. T. & Raymond, C. A. (eds) in *The Dawn Mission to Minor Planets 4 Vesta and 1 Ceres*, 3-23 (Springer, 2012).

[25] Harmon, J. K. in *Mercury* (eds Balogh, A., Ksanfomality, L. and von Steiger, R.) 125-167 (Springer, 2008).

[26] Simpson RA (2011). Polarization in bistatic radar probing of planetary surfaces: application to Mars Express data. Proc. IEEE.

[27] Simpson RA (1993). Spacecraft studies of planetary surfaces using bistatic radar. IEEE Trans. Geosci. Remote Sens..

[28] Krening, S. C., Semenov, B. V. & Acton, C. H. Dawn SPICE kernels v1.0, DAWN-M/A-SPICE-6-V1.0, NASA Planetary Data System (2012).

[29] Mitchell DL (1996). Radar observations of asteroids 1 Ceres, 2 Pallas, and 4 Vesta. Icarus.

[30] Palmer EM, Heggy E, Capria MT, Tosi F (2015). Dielectric properties of asteroid Vesta’s surface as constrained by Dawn VIR observations. Icarus.

[31] Williams DA, Yingst RA, Garry WB (2014). Introduction: the geologic mapping of Vesta. Icarus.

[32] Reddy V (2012). Delivery of dark material to Vesta via carbonaceous chondritic impacts. Icarus.

[33] Heggy E (2012). Radar properties of comets: parametric dielectric modeling of Comet 67P/Churyumov-Gerasimenko. Icarus.

[34] Hérique A (2016). Cosmochemical implications of CONSERT permittivity characterization of 67P/CG. Mon. Not. R. Astron. Soc..

[35] Scully JEC (2014). Geomorphological evidence for transient water flow on Vesta. Earth Planet. Sci. Lett..

[36] Thompson TW, Masursky H, Shorthill RW, Tyler GL, Zisk SH (1973). A comparison of infrared, radar and geologic mapping of lunar craters. The Moon.

[37] Chen C-C (2000). Small Deep Space Transponder (SDST) DS1 validation report. NASA Deep Space 1 Technology Validation Reports JPL Publication.

[38] Tyler GL (2001). Radio science observations with Mars Global Surveyor: orbit insertion through one Mars year in mapping orbit. J. Geophys. Res..

[39] Preusker, F. et al. Dawn FC2 derived Vesta DTM SPG v1.0, DAWN-A-FC2-5-VESTADTMSPG-V1.0, NASA Planetary Data System (2016).

[40] Taylor, J. (ed), Fernández, M. M., Bolea-Alamañac, A. & Cheung, K.-M. in *Deep Space Communications*, 139–200 (John Wiley & Sons, 2016).

[41] Ogilvy, J. A. (ed.) in *Theory of Wave Scattering From Random Rough Surfaces* 83-84 (Adam Hilger, 1991).

[42] Thomas PC (1997). Impact excavation on Asteroid 4 Vesta: Hubble Space Telescope results. Science.

[43] Kennedy, B. et al. in *Spaceflight Mechanics 2013* (eds Tanying, S., Park, R. S., Starchville, Jr., T. F. & Newman, L. K.) *Advances in Astronautical Sciences* **148**, 2251–2270 (Univelt, 2013).

