# Publisher Correction: Orbital bistatic radar observations of asteroid Vesta by the Dawn mission

**DOI:** 10.1038/s41467-017-01617-x

**Published:** 2017-11-23

**Authors:** Elizabeth M. Palmer, Essam Heggy, Wlodek Kofman

**Affiliations:** 1 Department of Geosciences, Western Michigan University, 1903 W. Michigan Avenue, Kalamazoo, MI 49008-5241 USA; 2 Ming Hsieh Department of Electrical Engineering, University of Southern California, 3737 Watt Way, Los Angeles, CA 90089 USA; 3 Jet Propulsion Laboratory, California Institute of Technology, 4800 Oak Grove Dr., Pasadena, CA 91109 USA; 4 UGA/CNRS, Institut de Planétologie et d’Astrophysique de Grenoble (IPAG) UMR 5274, Grenoble, F-38041 France; 5 Space Research Centre of Polish Academy of Sciences, Bartycka 18A, 00-716 Warsaw, Poland

*Nature Communications* **8**:409 doi:10.1038/s41467-017-00434-6; Article published online 12 September 2017

An incorrect version of the Supplementary Information was inadvertently published with this Article. The HTML has now been updated to include the correct version of the Supplementary Information.

