# Supplementary material for: Orbital bistatic radar observations of asteroid Vesta by the Dawn mission
Source: Nat Commun. 2017 Sep 12;8:409. doi: 10.1038/s41467-017-00434-6 (PMC5595829; doi:10.1038/s41467-017-00434-6)
Supplement: Supplementary file 1 — Supplementary Information [file 41467_2017_434_MOESM1_ESM.pdf]

Supplementary Table 1. Dawn bistatic radar acquisition parameters at asteroid Vesta.

Transmitter<br>Dawn Spacecraft High-Gain Communications Antenna		
Power P_t	100 ± 0.03 W	[1]
Wavelength λ	3.55 cm	[1]
Frequency f	8.435 GHz (X-Band)	[1]
Polarization	RCP	[1]
Gain G_t	39.60 ± 0.25 dBi	[1]
Total loss L:	–2.20 dB ± 0.10 dB	
HGA transmit circuit loss	–0.41 ± 0.057 dB	[1]
HGA degrees-off-boresight loss	–1.40 ± 0.026 dB	[1]
Atmospheric attenuation loss	–0.07 ± 0.00 dB	[1]
DSN 70-m antenna pointing loss	–0.10 ± 0.057 dB	[2]
DSN 70-m polarization loss	–0.20 ± 0.057 dB	[2]
FWHM beamwidth θ_{bw}	~1.6°	[3]
Angle of incidence θ_i	~89°	
Receiver<br>Earth Deep Space Network 70-m Antennas		
Gain G_r	74.6 ± 0.2 dBi	[4]
Polarization	RCP and LCP	[4]
Sampling rate	16,000 samples/s	
Total System Noise Temperature T_{sys}:	22.9 ± 1.6 K	
T_{sys} in vacuum at zenith	11.7 ± 1.4 K	[4]
due to elevation (lowest limit, 25°)	7 K	[4]
due to atmosphere	3.3 ± 1.2 K	[4]
due to Sun	0.89 K	[1]
due to other hot bodies	0.00 K	[1]

Supplementary Table 2. Instantaneous velocities of Dawn (D), the echo site on Vesta (Vpt) and the receiving antenna on Earth (E) during occultation entry, orbit 355 at 03:47:06 UTC.

Component of velocity vector	v_D (m s^{-1})	v_{Vpt} (m s^{-1})	v_E (m s^{-1})
Total in bistatic plane	84.4	96.5	39600
$\hat{r}_D$	–	-86.7	-20396
$\hat{r}_{Vpt}$	-82.2	–	-20396
$\hat{r}_E$	-82.6	86.0	–

v_A : velocity (positive when moving toward the target) — $\hat{r}_B$: radial unit vector from body A toward target B

Supplementary Table 3. Individual contributions of the differential Doppler shift δf_{total} between the surface echo and direct signal due to motion of the Earth-based receiving antenna (Δf_{direct}), the motion of the Dawn spacecraft (δf_{orbit}) and Vesta's rotation ($\delta f_{rotation}$).

Δf_{direct}	δf_{orbit}	$\delta f_{rotation}$	δf_{total}
$\frac{1}{\lambda}(v_D \; \hat{r}_E + v_E \; \hat{r}_D)$	$\frac{1}{\lambda}(v_D \; \hat{r}_{Vpt}) - \Delta f_{direct}$	$\frac{1}{\lambda}(v_{Vpt} \; \hat{r}_D + v_{Vpt} \; \hat{r}_E) - \Delta f_{direct}$	$\delta f_{orbit} + \delta f_{rotation}$
–584,434 Hz	–9 Hz	+7 Hz	–2 Hz

**Negative differential Doppler shifts indicate a decrease in frequency (motion away from the observer), such that the surface echo reflection has a greater path length to Earth than the direct signal during occultation entry.*

Supplementary Table 4. Sources of uncertainty in the calculation of theoretical δf and σ (km^2).

Parameter	Uncertainty	Description
Position of Dawn r_D	± 3 m	Uncertainty in LAMO reconstructed trajectory [5]
Radius of echo site R_{Vpt}	± 0.5 km	Due to variation in topography within the large radar-illuminated echo site
Latitude of echo site center	± 0.2°	Estimated uncertainty
Longitude of echo site center	± 0.2°	Estimated uncertainty
Measured surface echo power $P_{r\,echo|meas}$	± 0.17 $\overline{P_{N\,echo}}$	Due to standard deviation of noise power
Measured direct power $P_{r\,dir|meas}$	± 0.17 $\overline{P_{N\,dir}}$	Due to standard deviation of noise power

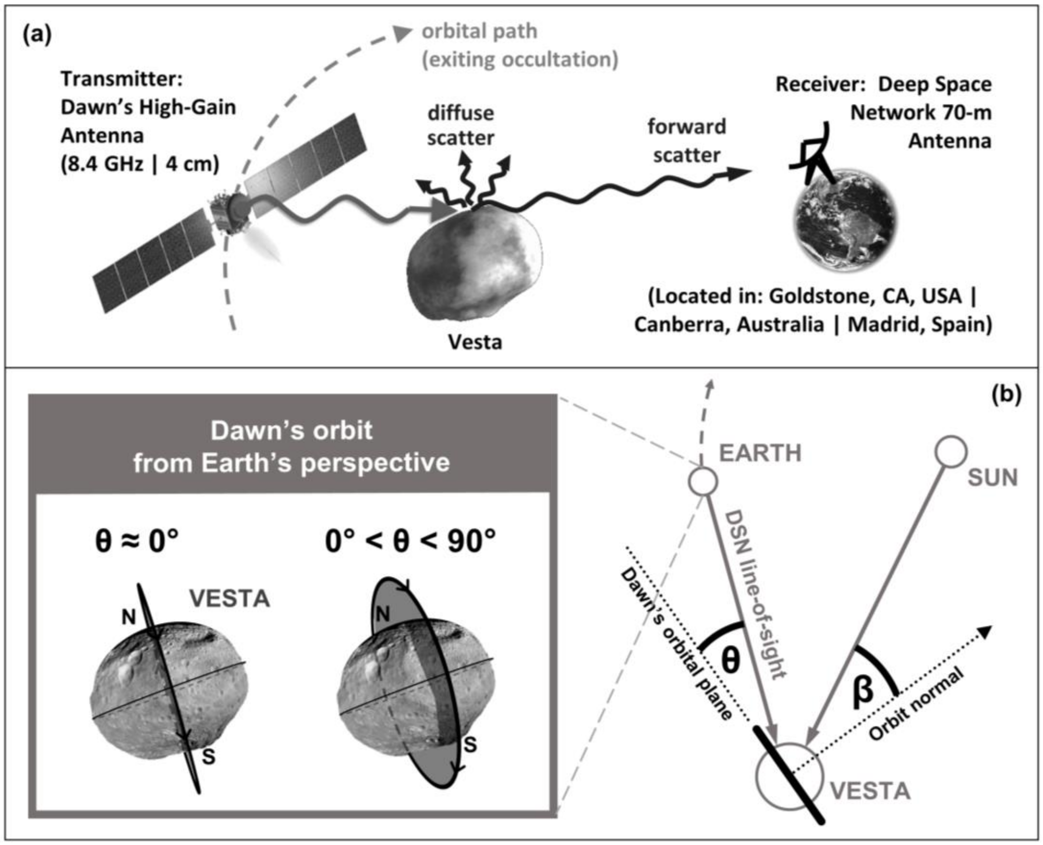

Supplementary Figure 1. Typical orbital geometry of Dawn spacecraft during bistatic radar observations of Vesta (not to scale). In panel (a), Dawn's HGA transmits radio waves during exit from occultation, which then scatter in all directions from Vesta's surface at the echo reflection site. Forward-scattered radio waves are then received at one of the DSN antennas on Earth. Panel (b) illustrates surface reflections occur from mid-latitudes on Vesta even though Dawn is in a polar orbit. Angle β is fixed at 45° to ensure that solar panels are under constant illumination and to maximize the visibility of Vesta's sunlit surface for spectroscopic and framing camera (FC) imaging. From the view of Earth-based receiving antennas, Dawn's orbital plane is tilted by angle θ that varies with the orbits of Earth and Vesta around the Sun, such that $0° < \theta < 90°$. Since Dawn's HGA is constantly pointed at Earth, surface radar reflections occur along mid-latitudes rather than from Vesta's poles.

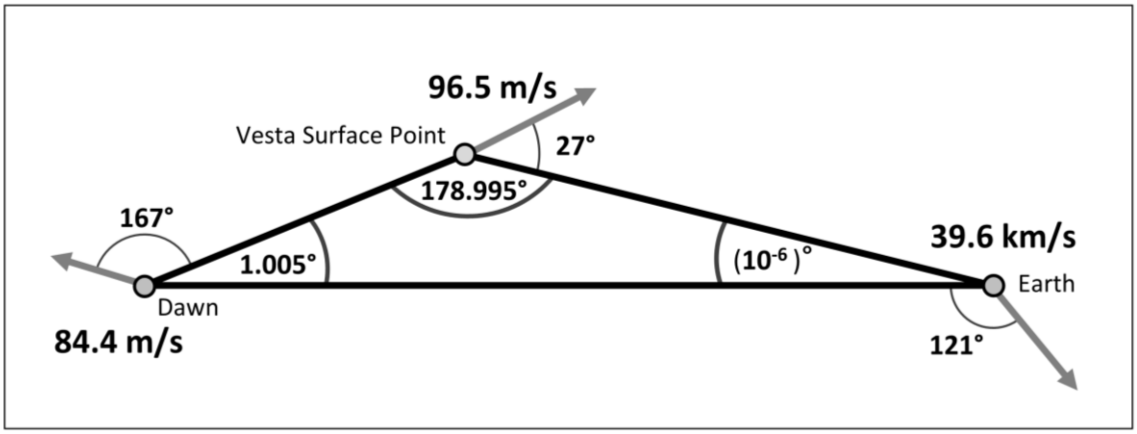

Supplementary Figure 2. Relative motion of Dawn's transmitting antenna, the Earth-based receiver, and the point of reflection on Vesta's surface within the bistatic plane during occultation entry of orbit 355. Velocities are expressed in Vesta's inertial frame of reference. The theoretical relative Doppler shift between the surface echo and direct signal ranges from ~2 to 20 Hz.

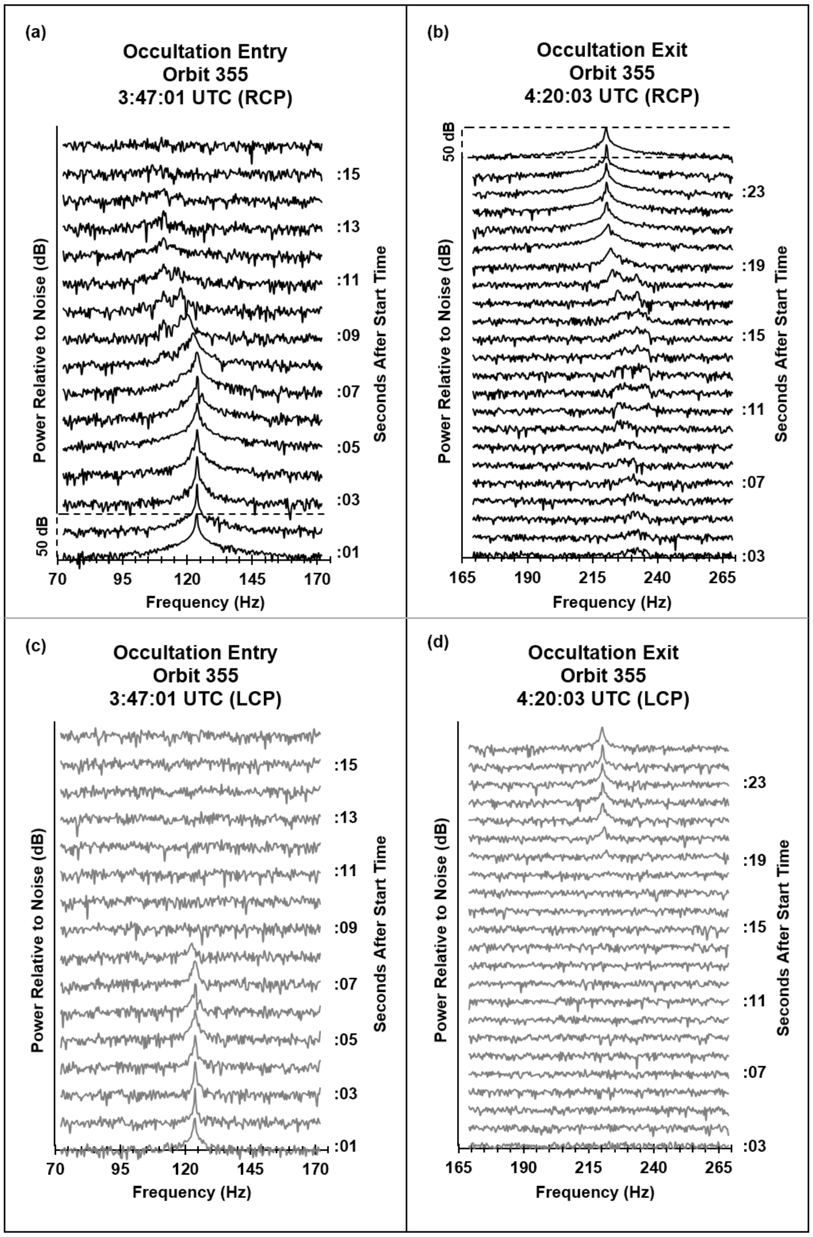

Supplementary Figure 3. Received signal in right- and left-hand circular polarization (RCP and LCP) during (a, c) occultation entry and (b, d) exit during orbit 355. At grazing incidence, surface radar reflections are in the regime of forward scattering. Hence, polarization is conserved such that there is no measurable LCP component in surface radar reflections from Vesta. The presence of LCP power (c, d) in the direct signal is the result of imperfection in the transmitting antenna and the high sensitivity of the DSN receiving system. At its maximum strength, the LCP component of the direct signal is 36 dB above the noise level (i.e. 2.6% of the power measured in RCP).

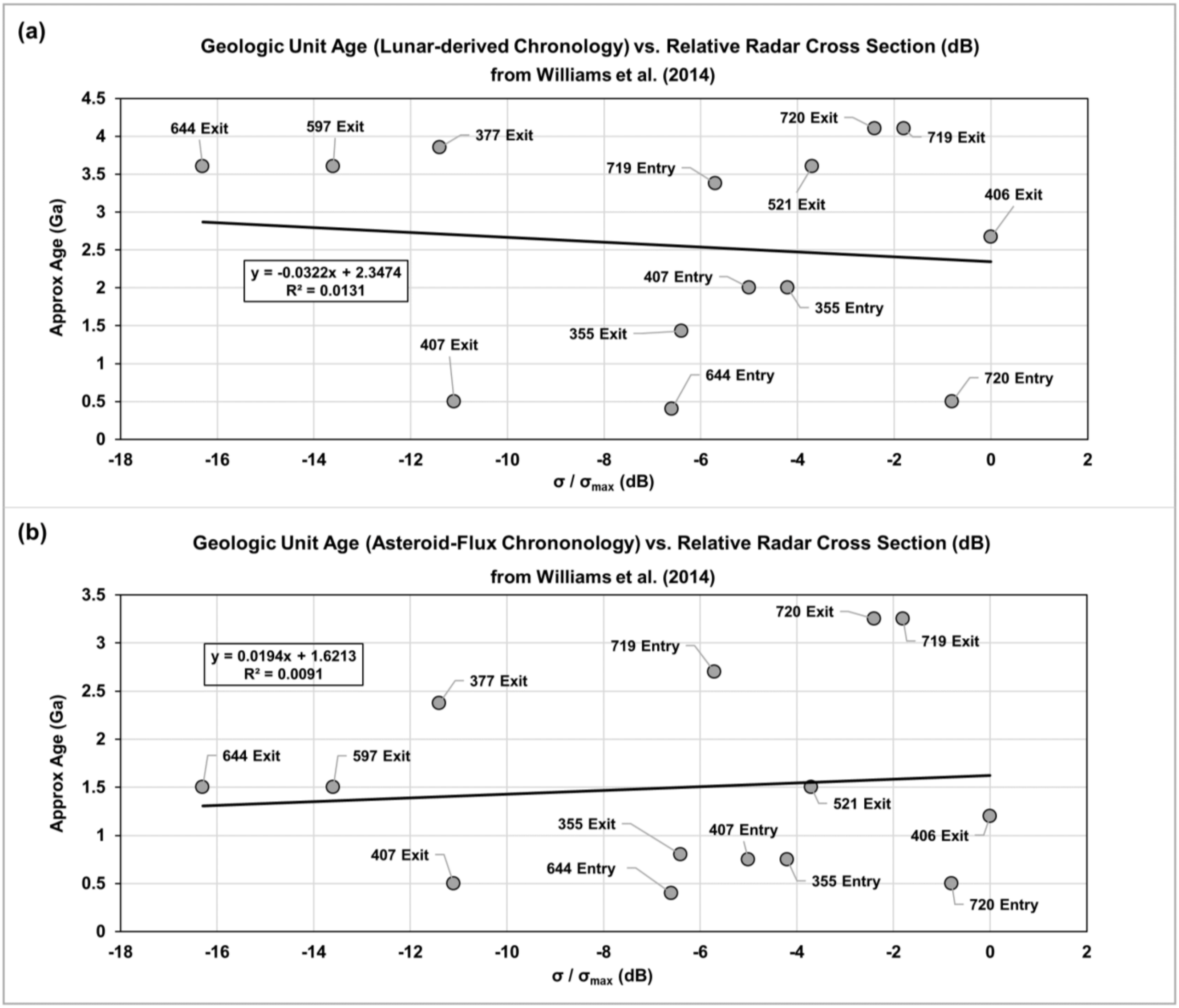

Supplementary Figure 4. Comparison of geologic unit age [6] with radar forward-scattering properties at each echo site on Vesta. Panel (a) plots geologic unit ages derived from lunar chronology while plot (b) shows surface ages derived from asteroid-flux chronology. Relative radar forward-scatter cross section (σ/σ_{max}) is calculated for each surface echo with respect to the site of strongest surface echo reflection (occultation exit of orbit 406). Since strong reflections are interpreted to come from smooth surfaces, lower values of (σ/σ_{max}) suggest rougher surfaces than the reference site. Unlike the Moon, Vesta does not exhibit a strong correlation between centimeter-to-decimeter-scale radar scattering properties and surface age, as is shown by the low correlation factor *R*.

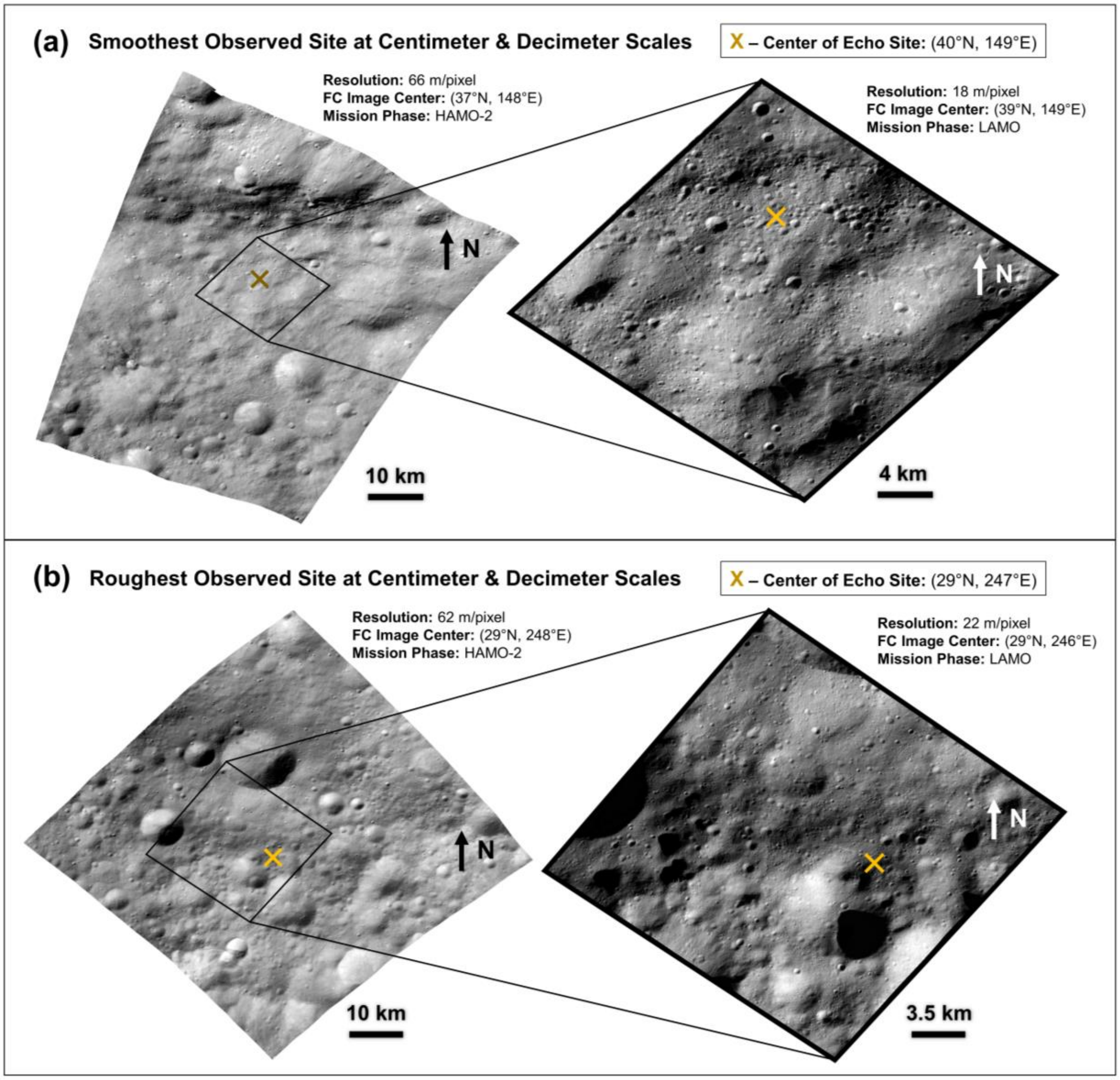

Supplementary Figure 5. High-resolution Dawn framing camera (FC) images of the echo sites with the strongest and weakest radar reflectivity. Roughness appears nearly equal at the resolution of the FC images, whereas radar reflectivity of each site suggests that (a) is much smoother than (b) at centimeter to decimeter scales (below the FC resolution). Images to the left cover the approximate extent of echo sites at ~65-m/pixel resolution acquired during one of Dawn's high-altitude mapping orbits (HAMO or HAMO-2) around Vesta. Images on the right show a subset of echo sites at ~20-m/pixel resolution acquired during Dawn's lowest-altitude mapping orbit (LAMO) of Vesta, where the center of each echo site is marked by an X. Radar-wavelength scale surface roughness on Vesta does not appear to correlate with surface topography at the scale of meters to tens of meters, indicating that cratering alone cannot dictate Vesta's surface texture.

Supplementary References

1. Taylor, J. Dawn Telecommunications, *NASA DESCANSO Design and Performance Summary Series* Article 13, 1-68 (2009).

2. Taylor, J., Fernández, M. M., Alamanac, A. I. B. & Cheung, K.-M. Deep Space 1 Telecommunications, *NASA DESCANSO Design and Performance Summary Series* Article 2, 1-63 (2001a).

3. Taylor, J., Cheung, K.-M. & Wong, C.-J. Mars Global Surveyor Telecommunications, *NASA DESCANSO Design and Performance Summary Series* Article 1, 1-40 (2001b).

4. Slobin, S. D. in *DSN Telecommunications Link Design Handbook (810-005),* Space Link Interfaces, Module 101, Rev. F. Retrieved from https://deepspace.jpl.nasa.gov/dsndocs/810-005/spacelink.cfm (2015).

5. Krening, S. C., Semenov, B. V. & Acton, C. H. Dawn SPICE Kernels V1.0, DAWN-M/A-SPICE-6-V1.0, NASA Planetary Data System (2012).

6. Williams, D. A. et al. The chronostratigraphy of protoplanet Vesta, *Icarus* **244**, 158-165 (2014).
